# Population Pharmacokinetics of Caspofungin and Dosing Optimization in Children With Allogeneic Hematopoietic Stem Cell Transplantation

**DOI:** 10.3389/fphar.2020.00184

**Published:** 2020-03-02

**Authors:** Chang-He Niu, Hua Xu, Liu-Liu Gao, Ying-Ming Nie, Li-Peng Xing, Li-Peng Yu, San-Lan Wu, Yang Wang

**Affiliations:** ^1^Department of Clinical Pharmacy, Wuhan Children’s Hospital, Wuhan Maternal and Child Healthcare Hospital, Tongji Medical College, Huazhong University of Science & Technology, Wuhan, China; ^2^Department of Cell Therapy and Transplantation Medicine, Wuhan Children’ Hospital, Wuhan, China; ^3^Department of Pharmacy, Union Hospital, Tongji Medical College, Huazhong University of Science & Technology (HUST), Wuhan, China

**Keywords:** caspofungin, pharmacokinetics, allogeneic hematopoietic stem cell transplantation, dosing, children

## Abstract

Caspofungin is the first echinocandin antifungal agent that licented for pediatric use in invasive candidiasis and aspergillosis. In this study, we evaluated the population pharmacokinetics of caspofungin and investigate appropriate dosing optimization against *Candida* spp. in children with allogeneic hematopoietic stem cell transplantation (allo-HSCT) in order to improve therapeutic efficacy. All participants received a recommended caspofungin 70 mg/m^2^ loading dose followed by 50 mg/m^2^ maintenance dose. A one-compartment model with first-order elimination was best fitted the data from 48 pediatric patients. Body surface area and aspartate aminotransferase had significant influence on caspofungin clearance from covariate analysis. Our results reviewed that dose adjustment is not necessary in patients with mild liver dysfunction. Monte Carlo simulations were performed using pharmacokinetic data from our study to evaluate the probability of target attainment (PTA) of caspofungin regimen in terms of AUC_24_/MIC targets against *Candida* spp. The results of simulations predicted that a caspofungin 70 mg/m^2^ at first dose, 50 mg/m^2^ of daily dose may have a high probability of successful outcome against *C. albicans* and *C. glabrata* whilst 60 mg/m^2^ maintenance dose was required for fungistatic target against *C. parapsilosis* but may be not sufficient to achieve optimal fungicidal activity. Caspofungin standard regimen had high probability of successful outcome against *C. albicans* (MIC ⩽ 0.25 mg/L) and *C. glabrata* (MIC ⩽ 0.5 mg/L) but insufficient for *C. parapsilosis* with MIC > 0.25 mg/L. That may provide an evidence based support to caspofungin individualized administration and decrease the risk of therapeutic failure in allo-HSCT pediatric patients.

## Introduction

Invasive fungal infections (IFIs) remains life-threatening in pediatric patients with immunocompromised status, such as those who receiving allogeneic hematopoietic stem cell transplantation (allo-HSCT), receipt of systemic corticosteroids, or bone marrow transplantation (Eggimann et al., 2003; Nivoix et al., 2008). These diseases cause considerable mortality and morbidity in pediatric patients. Invasive candidiasis and invasive aspergillosis are responsible for IFIs, the case fatality rates estimate to be 30% and 50%.

Caspofungin, the first echinocandin antifungal agent to become licensed for use in pediatric patients (3 months of age and older) by the USA for the treatment of invasive candidiasis, invasive aspergillosis, or as empirical therapy for presumed fungal infections (Sable et al., 2008; VandenBussche and Van Loo, 2010). Fewer drug interactions and adverse effects make the echinocandins safe to administer (Pappas et al., 2016). Caspofungin has the largest number of FDA indications among the echinocandin class of drugs. However, dosage regimens of caspofungin in pediatric patients cannot be consistent with adults in immune response and drug disposition. Drug exposure can be significantly influenced by maturation of renal and metabolic function occurs rapidly especially in young infants (Castro et al., 2017). There is a higher incidence of nosocomial infection especially the fungal infection in children patients at early stage undergoing allo-HSCT. A reasonable antibiotics and preventing the fungal infection are good to control nosocomial infection in children treating with allo-HSCT. Caspofungin is an effective strategy for preventing IFIs. Dosing adjustment is necessary in pediatric patients for caspofungin to maximize favorable outcomes. Therefore, the purpose of this study is to evaluate the population pharmacokinetic (PopPK) parameters of intravenous caspofungin and then establish an evidence-based individualized administration use of caspofungin in pediatric patients with allo-HSCT.

## Materials and Methods

### Study Design

A prospective, open-label pharmacokinetic study of caspofungin was performed at Wuhan Children's Hospital, Hubei Province. All designed procedures conformed to the legal requirements and the ethics committee of Wuhan Children's Hospital approved this study.

Caspofungin has been approved for use in patients with IFI that requiring prevention treatment, especially for patients undergoing allo-HSCT. In total, 48 patients, received allo-HSCT, fulfilled the enrollment criteria and provided informed in the model building group between July 2017 and March 2019. They received caspofungin as a 1-h intravenous infusion on the basis of body surface area (BSA) with 50 mg/m^2^ maintenance dose daily followed by 70 mg/m^2^ loading dose on day 1.

### Pharmacokinetic Sampling

We performed an opportunistic sampling strategy when a steady-state caspofungin concentration had been achieved. Plasma was placed on ice and centrifuged (15,000 rpm for 5 min at +4°C) immediately and then stored at −80°C prior to analysis. Then we recorded the dosing and infusion time as well as sampling time prospectively.

### Analytical Methods for Caspofungin

Caspofungin concentrations were detected by a high-performance liquid chromatography method (Agilent Technologies Inc., 1260 infinity) with ultraviolet (UV) performed on detection by fluorescence. The excitation wavelength was 220 nm, the column temperature was 30 °C, and the pump flow rate was 0.8 ml/min. A solution of 42% acetonitrile was used as the mobile phase. We employed 0.5 ml of serum loaded on solid-phase extraction column (Agela Technologies, Cleanert ODS C18, 200 mg/3 ml) and then eluted with 60% acetonitrile. An Innoval C18 column (Agela Technologies, 10 μm, 4.6 × 250 mm) was used for separation. The calibration curve ranged from 0.6 to 20 mg/L. The inter- and intra-assay coefficients of variation of controls were all less than 5%. The lower limit of quantification (LLOQ) was 0.6 mg/L.

### Population Pharmacokinetic Modeling of Caspofungin

The PopPK study was performed by applying the non-linear mixed-effect modeling program Phoenix NLME (Version 8.1, Pharsight Corporation) and R program (Version 3.5.1, http://www.r-project.org/).

We tried to describe the pharmacokinetic process of caspofungin *in vivo* by using the one- or two-compartment models. The scale model and exponential model were adopted for intra-individual variation and inter-individual variation respectively. A forward and backward selection process was used for covariate analysis. The effects of demographic characteristics including age, body weight (WT), height, BSA, biological parameters including uric acid (UA), serum cystatin C (Cys-C), estimated glomerular filtration rate (eGFR), alanine aminotransferase (ALT), aspartate aminotransferase (AST), and gama-glutamyltranspeptidase (gama-GT) that were investigated as potential covariates on PK parameters. eGFR was calculated according to the modified Schwarta formula (Schwartz et al., 2009). Mosteller formula was used to calculate the body surface area (BSA) as follows (Yang et al., 2019):

$$\text{BSA}({\text{m}}^{2})={\left[\text{height}(\text{cm})\times \text{weight}(\text{kg})/3600\right]}^{1/2}$$

During the forward selection step, the covariate causing the objective function value (OFV) decreased more than 3.84 (P < 0.05, χ2 distribution, df = 1), the covariate was included into the model, thereafter we tested the remaining covariates. A stepwise backward elimination procedure was carried out once no more covariates resulted in a significant OFV reduction. If the removal of the covariate resulted in an increased OFV less than 6.65 (P < 0.01, χ2 distribution, df = 1) the covariates should be remained in the final PK model.

In this study, goodness-of-fit plots, bootstrap, visual predictive check (VPC), and normalized prediction distribution errors (NPDEs) were adopted to model validation. Bootstrap resampling 1,000 times in the modeling data, and the model was established with the sampling data set. We compared the similarity between bootstrap model parameters and the parameters in final model. VPC simulated 1,000 virtual data with the final model and analyze whether the 95% confidence interval (CI) of the simulated data contains the original data. NPDEs also adopted simulation technology and simulated 1,000 times based on the final model to investigated the distribution of the standard error and perform the normal distribution test.

### Dosing Regimen Evaluation and Optimization Based on the Pharmacokinetic Model

The likely success of treatment identified with the probability of target attainment (PTA) by comparing the pharmacodynamic exposure against an minimal inhibitory concentration (MIC) distribution. Previous pharmacodynamic studies have demonstrated that the 24 h area under the concentration-time curve (AUC_24_)/MIC was a good marker of the caspofungin exposure-response relationship (Bellman et al., 2017). A PK/PD target was defined as the ratio AUC_24_/MIC for candidiasis. Fungicidal target AUC_24_/MIC ratios were 865 for *C. albicans*, 450 for *C. glabrata*, and 1,185 for *C. parapsilosis*. Fungistatic target AUC_24_/MIC ratios of caspofungin for *C. albicans*, *C. glabrata*, and *C. parapsilosis* are 748, 96.2, and 559 respectively (Andes et al., 2010).

Two simulation procedures were performed to calculate the probability of PK/PD target attainment with different dosing regimens based on the final model. One of the procedure is fixed MIC value of *Candida* app. to simulated the clinical efficacy of the different drug regimens of caspofungin in infants, children (age 2–11 years), and adolescents (age 12–14 years). According to the literature report, the MIC_90_ value for *C. albicans, C. glabrata*, and *C. parapsilosis* are 0.03, 0.06, and 0.5 mg/L (Pfaller et al.). Another simulation procedure is not fixed MIC values. The loading doses of caspofungin 50, 60, 70, and 80 mg/m^2^ and maintenance doses 40, 50, 60, and 70 mg/m^2^ were simulated for *C. albicans*, *C. glabrata*, and *C. parapsilosis* with different MICs (range 0.015–2.0 mg/L).

## Results

### Study Population

Forty-eight patients were included in this study. The mean (SD; range) age and body weight were 6.58 (3.7; 0.6–14) years and 21.7 (10.3; 7.5–54.0) kg, respectively. During the caspofungin therapy, no patients discontinued and all patients fulfilled the inclusion criteria. The mean (SD; range) BSA of all 48 children in this study was 0.80 m^2^ (0.27, range 0.38–1.50). There were 37 children with normal hepatic function (AST ⩽ 40 U/L), nine children with mild hepatic dysfunction (40 U/L < AST ≤ 120 U/L), and two children with severe hepatic dysfunction (AST > 120 U/L). **Table 1** presented the demographic and clinical characteristics of participants.

**Table 1:** Baseline characteristics of 48 children.

	Number	Mean(SD)	Median(Range)
Patients	48		
Gender	31M:17F		
Samples	139		
Samples per patient		2.9 (0.6)	
Sampling time after the last dose (h)		17.5 (2.2)	18 (7.5–21)
Age (years)		6.58 (3.7)	6.2 (0.61–14)
Weight (kg)		21.7 (10.3)	20 (7.5–54)
Height (cm)		112.9 (23.6)	113 (69–160)
Body surface area (BSA) m^2^		0.8 (0.27)	0.8 (0.4–1.5)
BUN (mmol/L)		5.18 (4.11)	4.3 (1.6–23.2)
SCR (μmol/L)		0.45 (0.3)	0.3 (0.2–4.4)
UA (μmol/L)		206.2 (113.4)	187 (142.3–770)
Cys-C (mg/L)		1.2 (0.6)	1.02 (1.0–13.2)
eGFR (ml/min·1.73 m^2)^		125.6 (45.7)	126.2 (126–263.3)
TBIL (μmol/L)		15.4 (10.9)	12.9 (9.3–157.8)
AL T (U/L)		57.9 (184.6)	14 (14–5147)
AST (U/L)		82.5 (262.5)	26 (25–11123)
gama-GT (U/L)		25.8 (30.3)	15 (11–202)

Gender: M for male, F for female; Range: minimum value-maximum value; WT, current body weight; BSA, body surface area; BUN, blood urea nitrogen; SCR, serum creatinine concentration; UA, uria acid; Cyc-C, serum cystatin C; eGFR, estimated glomerular filtration rate; TBIL, total bilirubin; ALT, alanine aminotransferase; AST, aspartate aminotransferase; gama-GT, gama-glutamyltranspeptidase.

### Population Pharmacokinetic Model Building

In total, 139 caspofungin concentrations were available for PopPK modeling with a range from 4.5 to 17.4 mg/L. One hundred four samples were obtained at the time points between peak and trough concentrations. The other 35 of them were trough concentration.

**Figure 1:**
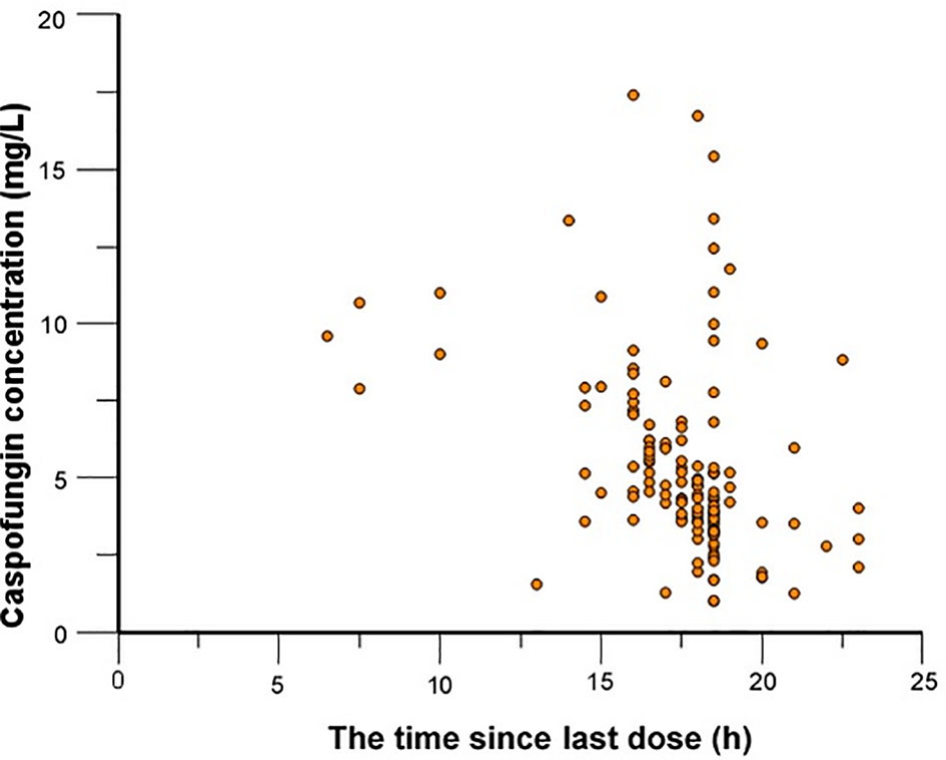
Caspofungin concentrations versus time.

**Figure 2:**
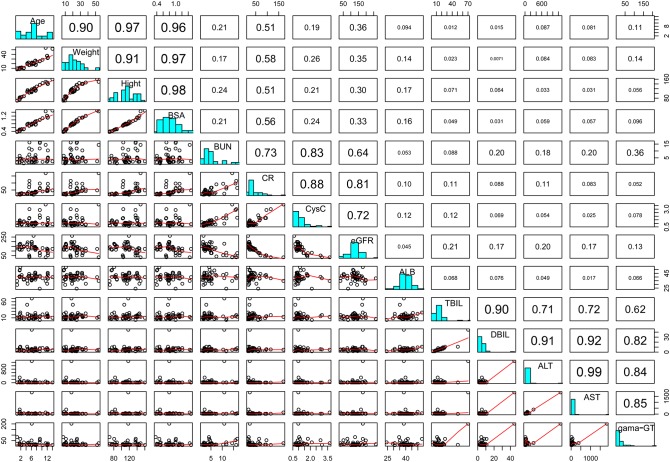
Correlation analysis of covariates.

Caspofungin plasma concentrations versus time profile was shown in **Figure 1**. Since the distribution phase plasma concentration data collected in this study are few, one compartment structural model with first order elimination was selected as the structural model. **Figure 2** showed covariates distribution and correlation analysis diagram. In order to avoid collinear programs, two covariates with a correlation coefficient greater than 0.5 are avoided to be included in the model simultaneously. An exponential model can best express the inter-individual variability, while a proportional model optimally describe residual variability. In the forward inclusion and backward-elimination procedure, BSA and AST retained in the final model as significant impacts on caspofungin clearance. Hypothesis test results of covariates affecting on caspofungin pharmacokinetic parameters are listed in **Table 2**.

**Table 2:** Hypothesis test results of clinical characteristics factors affecting on caspofungin pharmacokinetic parameters.

Hypothesis test	OFV	△OFV	P-value	Comments
Basic model	603.1			
Dose BSA affect CL?	563.5	−41.3	<0.01	YES
Dose weight affect CL?	591.8	−39.6	<0.01	YES
Dose CR affect CL?	600.8	−11.3	<0.01	YES
Dose eGFR affect CL?	602.7	−2.3	>0.05	NO
Dose ALB affect CL?	602.6	−0.4	>0.05	NO
Dose TBIL affect CL?	602.9	−0.5	>0.05	NO
Dose DBIL affect CL?	601.8	−0.2	>0.05	NO
Dose ALT affect CL?	578.6	−1.3	>0.05	NO
Dose AST affect CL?	599.9	−24.5	<0.01	YES
Dose gama-GT affect CL?	−453.5	−3.2	>0.05	NO

The final PopPK model formula is shown below:

$$CL\;(L\cdot h^{-1})\;=\;0.14\;\times \;{\left(BSA/0.79\right)}^{0.89}\times \;{\left(lnAST/3.38\right)}^{-0.23}$$

**Figure 3:**
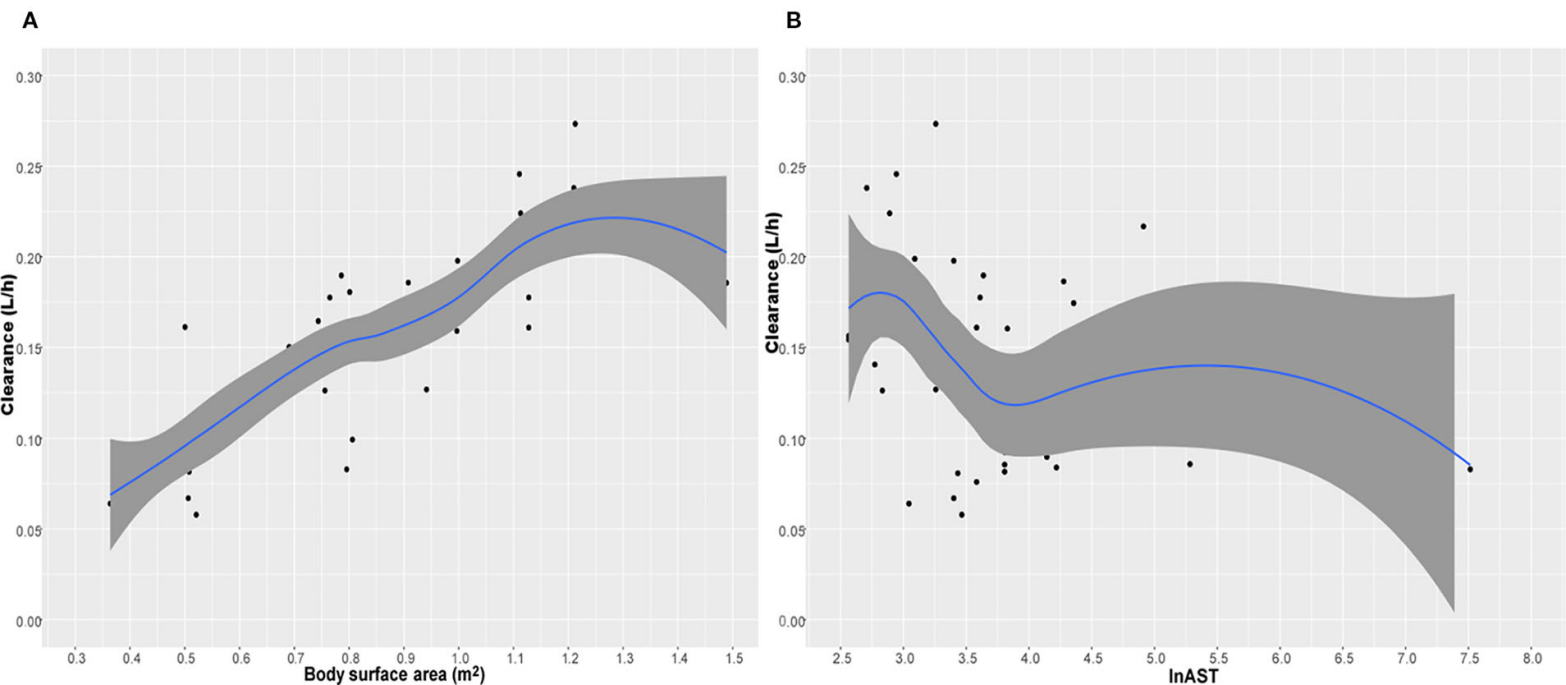
The relationship between caspofungin clearance (CL) versus body surface area (BSA) **(A)** and lnAST **(B)**. The shaded area represents 95% intervals for the locally weighted acatterplot smoothing (LOWESS) fit.

Vd(L) = 1.36 × (BSA/0.79)

where CL is the individual clearance, V_d_ is the individual volume distribution. **Table 3** displayed the population pharmacokinetic parameter estimates of the final PopPK model for caspofungin. Means ± SD Bayesian estimates BSA-normalized of CL and V_d_ were 0.19 ± 0.04 L/m^2^/h and 1.72 ± 0.01 L/m^2^, respectively. We used the locally weighted scatterplot smoothing (LOWESS) method to visualize the relationship between the defined covariates parameters and caspofungin CL (**Figure 3**). Caspofungin CL increased with BSA whereas decreased with lnAST that demonstrated by the LOWESS curves. Furthermore, according to the value of AST, patients were divided into three groups: normal liver function group, mild liver dysfunction, and severe liver dysfunction. Only two patients had severe liver dysfunction (AST>120 U/L) that being excluded from the study. Although the CLs of caspofungin were markedly lower in patients with mild liver dysfunction (40 U/L < AST ⩽ 120 U/L) as compared with normal liver function (0.117 ± 0.04 L/h vs 0.160 ± 0.05 L/h, P = 0.01). However, no statistical difference could be observed in estimated BSA-normalized CLs between the two groups (0.179 ± 0.03 L/m^2^/h vs 0.188 ± 0.03 L/m^2^/h, P = 0.45). It should be noted that dosage adjustment based on BSA is not necessary in pediatric patients with mild liver dysfunction. We also grouped the patients that excluded two severe liver dysfunction based on age: infants; children, age 2–11 years; adolescents, age 12–14 years. The BSA-normalized CLs of caspofungin were 0.190 ± 0.015 L/m^2^/h vs 0.187 ± 0.031 L/m^2^/h vs 0.178 ± 0.039 L/m^2^/h in the above age groups, respectively.

**Table 3:** Population pharmacokinetic parameters of caspofungin and bootstrap results.

Parameter	Final model		Bootstrap analysis			Bias
	Estimate	SE(%)	2.5^th^ percentile	Median Estimate	97.5^th^ percentile	(%)
θ_Vd_ (L)	1.36	15.58	0.75	1.34	1.87	−1.47
θ_CL_ (L·h^−1^)	0.14	8.50	0.10	0.14	0.17	0
θ_1_	0.89	11.36	0.62	0.91	1.20	2.25
θ_2_	−0.23	39.13	−0.49	−0.25	−0.01	−8.70
Inter-individual variability						
ω_Vd_(%)	32.9	14.5	23.5	32.9	42.3	0
ω_CL_(%)	33.3	21.3	19.5	35.1	50.8	5.4
Residual variability						
σ (%)	26.6	8.0	24.0	26.8	33.3	0.6

PopPK: population pharmacokinetic; SE(%), percent standard error; θ_Vd_,typical value of apparent clearance; θ_CL_, typical value of apparent volume of distribution; θ_1_, exponent for BSA as covariate for V_d_; θ_2_, exponent for lnAST as covariate for CL; ω_Vd_. square root of inter-individual variance for V_d_;ω_CL_, square root of inter-individual variance for CL; σ, residual variability Bias(%)= (Median Estimate Bootstrap – Estimate Final model)/Estimate Final model× 100%.

### Model Validation

**Figure 4:**
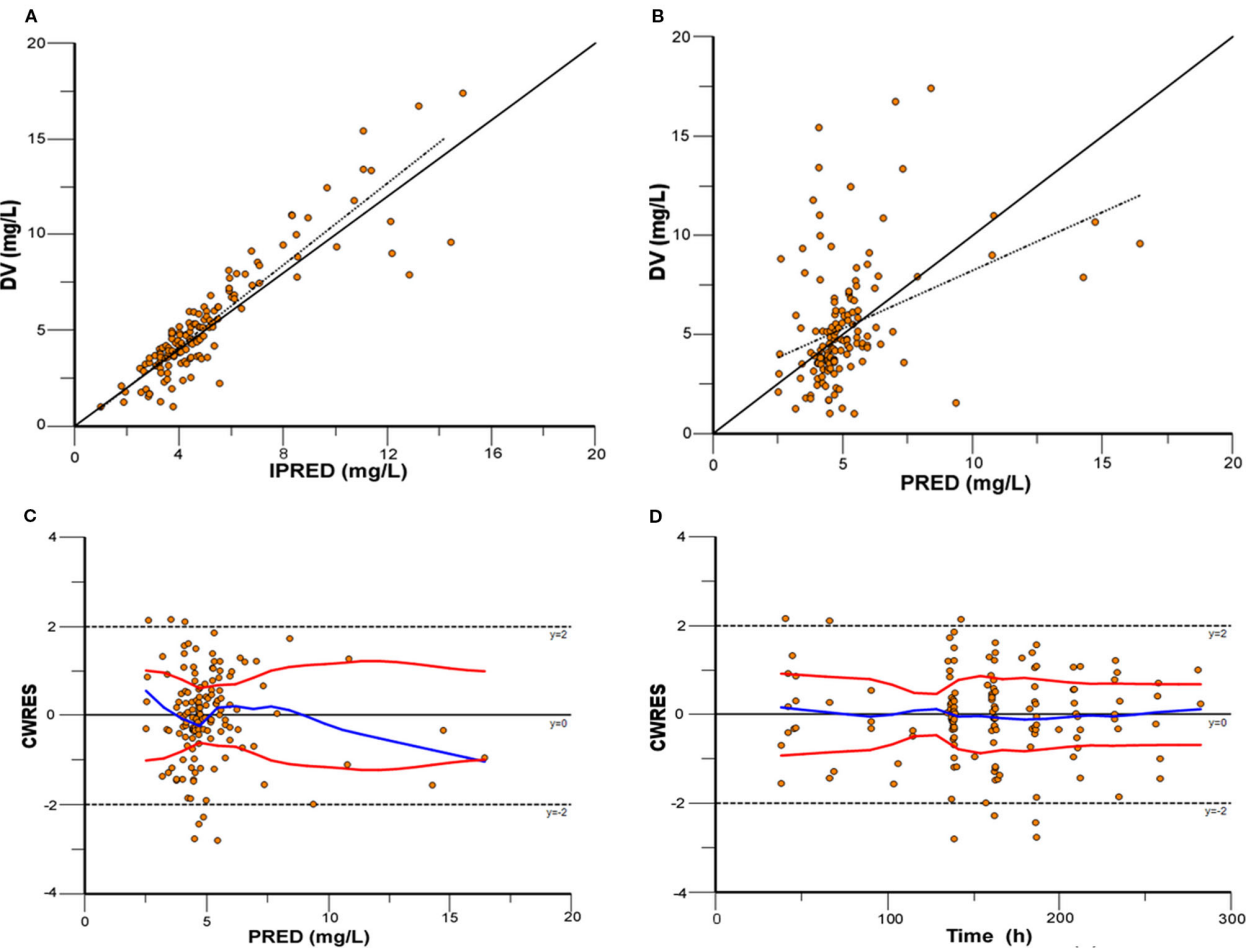
Model evaluation for caspofungin. **(A)** Observed concentrations (DV) versus individual prediction (IPRED); **(B)** DV versus population prediction (PRED); **(C)** Conditional weighted residuals (CWRES) versus PRED; **(D)** CWRES versus time.

**Figure 5:**
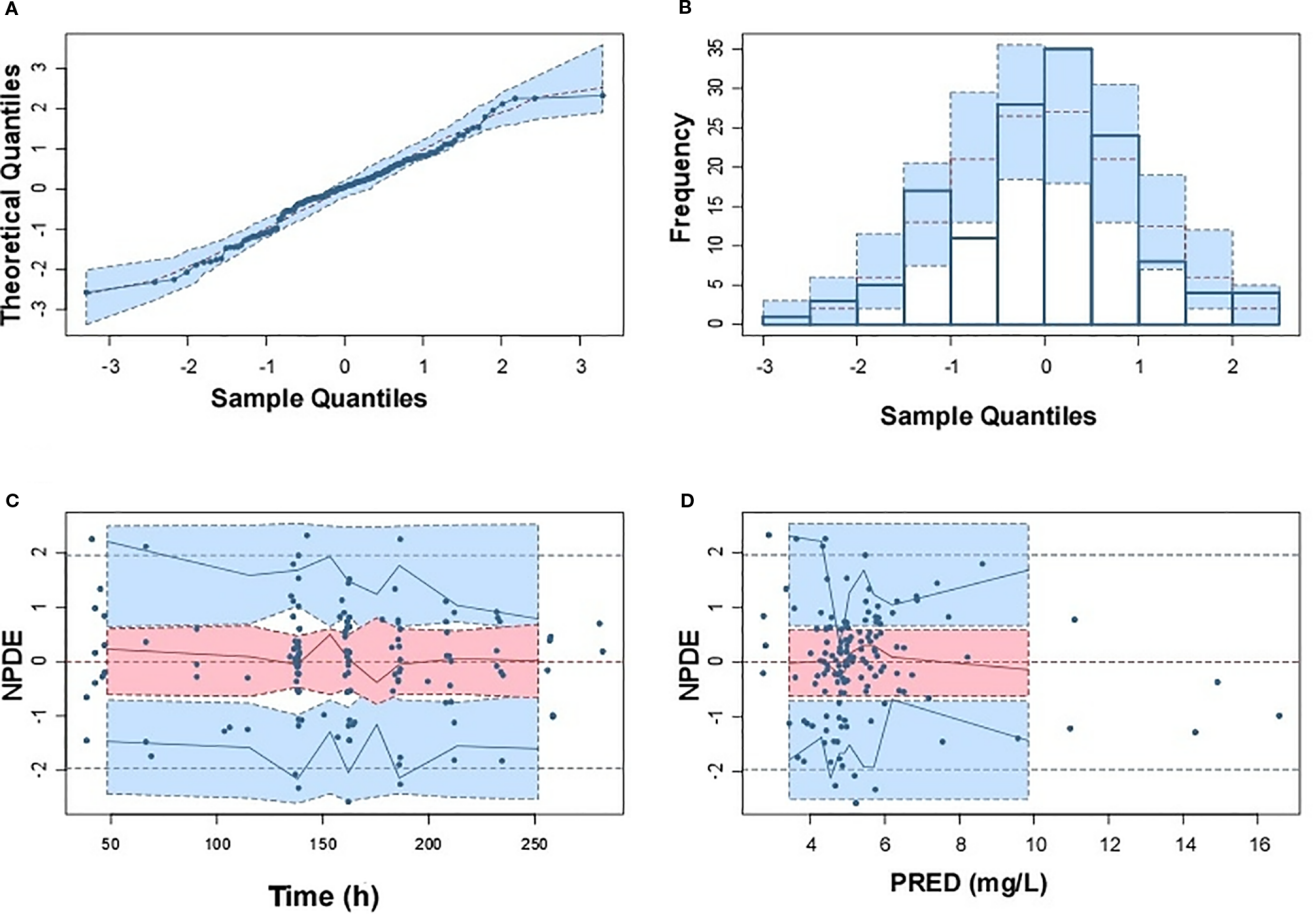
Normalized prediction distribution errors (NPDE) of the final population pharmacokinetic model. **(A)** Q-Q plot of NPDE versus the expected theoretical distribution; **(B)** Histogram of NPDE with the density of the standard normal distribution overlaid; **(C)** Scatterplot of NPDE versus time; **(D)** Scatterplot of NPDE versus population prediction (PRED).

**Figure 6:**
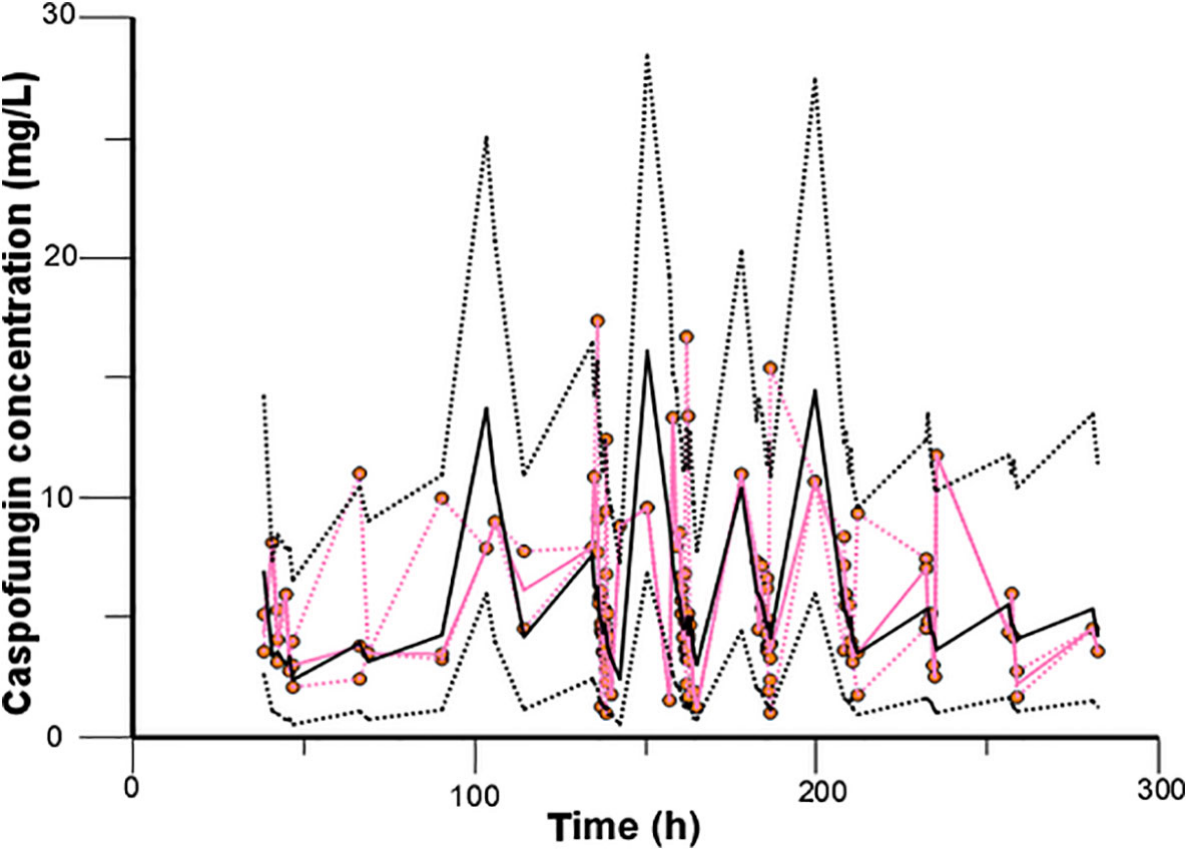
Validation of the model by the visual predictive check. The observed caspofungin concentrations are shown as orange circles. Red solid and dashed lines present the median, 2.5^th^ percentile, and 97.5^th^ percentile of the observed concentrations, and black solid and dashed lines represent the median, 2.5^th^ percentile, and 97.5^th^ percentile of the simulated concentrations.

Model diagnostics showed acceptable goodness-of-fit for the final model of caspofungin. From the visual biases that presented in **Figure 4**, the individual and population predictions were unbiased. **Table 3** indicated the median with 95% CI parameter estimates acquired from a 1,000-run bootstrap analysis. **Figure 5** displayed the results of NPDE analysis. The NPDE of an absolutely correct model should follow the normal distribution in theory. In this study, four tests were used to verify the NPDE distribution characteristics of the final model. The NPDEs followed a normal distribution and density, indicating a good fit of the model to the individual data. The VPCs plot (**Figure 6**) showed that the majority of the simulations were within the 95% CI of prediction, indicating no significant deviation between observed data and simulated data. Overall, the caspofungin PopPK model evaluation results revealed that the final model provided an adequate description of the data and a good prediction of individual PK parameters.

### Dosing Regimen Evaluation and Optimization

**Figure 7:**
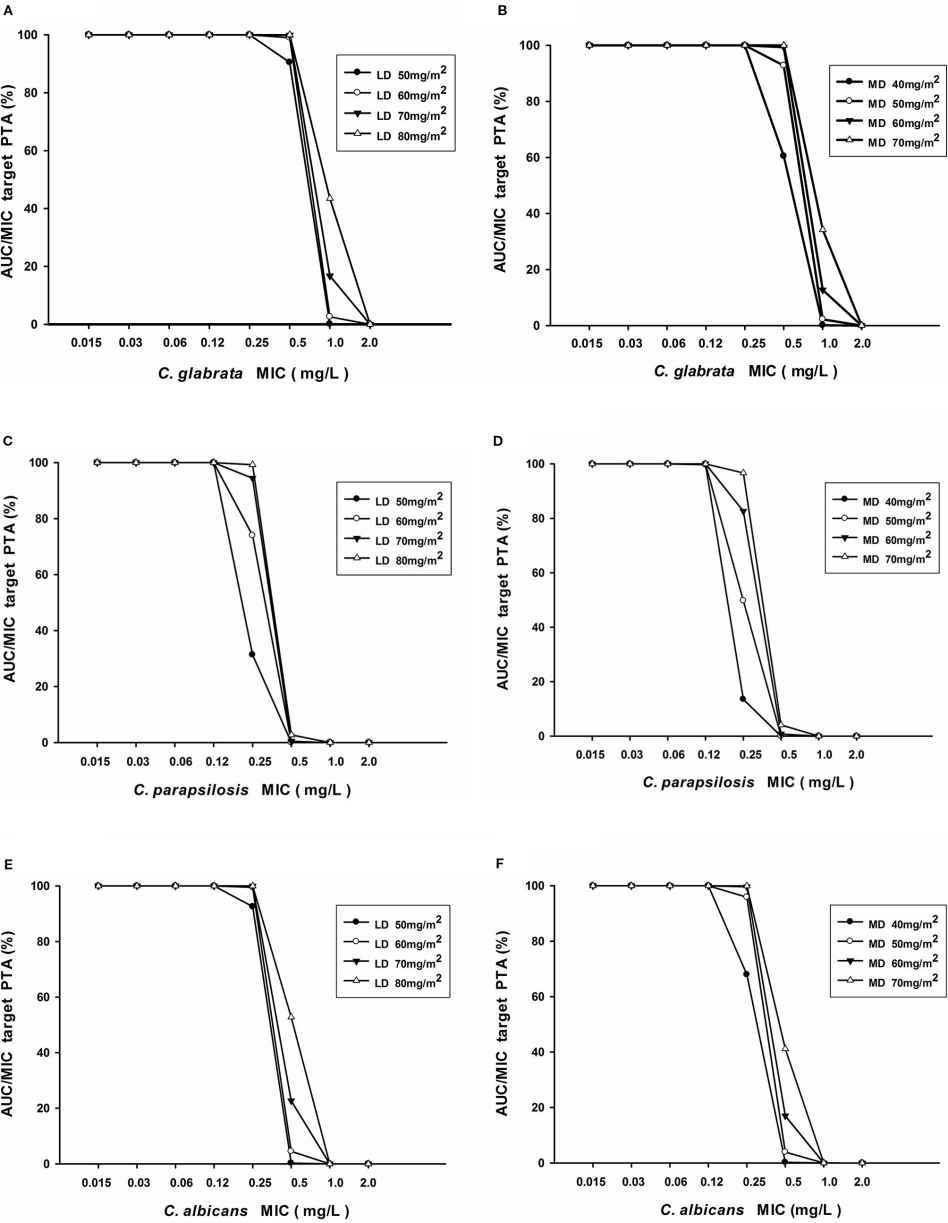
**(A–F)** Probability of target attainment of AUC_24_/MIC for various loading doses and maintenance doses of caspofungin in patients with *C. albicans*, *C. glabrata*, and *C. parapsilosis*.

**Table 4** displayed the assessment of PTA (%) value for different caspofungin dosage adjustment estimated according to AUC_24_/MIC against *C. albicans, C. glabrata*, and *C. parapsilosis*. Regarding *C. albicans* and *C. glabrata*, recommended caspofungin regimens (70 mg/m^2^ loading dose followed by 50 mg/m^2^ maintenance dose) can achieve the PTA value of ⩾ 90% for different age groups of pediatric patients. From the results of simulations, it predicted that 70 mg/m^2^ at first dose, 60 mg/m^2^ of daily dose for pediatric patients likely had an optimal (PTA ⩾ 90%) success of fungistatic target for *C. parapsilosis*. However, none of the dosage regimens of caspofungin achieved an expected fungicidal target. **Figures 7A–F** showed the PTA for various intravenous loading and maintenance doses of caspofungin against different *Candida* spp. MIC values. For *C. albicans* with an MIC ≤ 0.25 mg/L and *C. glabrata* with an MIC ≤ 0.5 mg/L, PTA over 90% best achieved with a loading dose of 50 mg/m^2^ and maintenance dose of 50 mg/m^2^ according to the PopPK analysis and Monte-Carlo simulations. However, 70 mg/m^2^ maintenance dose followed by 70 mg/m^2^ loading dose led to optimal achievement of germicidal therapeutic PK/PD targets that PTA ⩾ 90% for *C. parapsilosis* with MIC ⩽ 0.25 mg/L. When the MIC value is higher than the above, recommended caspofungin dosage regimens led to less successful rate in patients that were infected with *Candidiasis*.

**Table 4:** The area under the concentration-time curve (AUC)/minimal inhibitory concentration (MIC) target probability of target attainment (PTA) values(%) against *Candida albicans*, *Candida glabrata*, and *Candida parapsilosis* for caspofungin in pediatric patients.

Regimen	AUC/MIC target PTA(%)					
	*C. albicans* MIC_90_ = 0.04 mg/L		*C. glabrata* MIC_90_ = 0.06 mg/L		*C. parapsilosis* MIC_90_ = 0.5 mg/L	
	Fungistatic target	Fungicidal target	Fungistatic target	Fungicidal target	Fungistatic target	Fungicidal target
**Loading dose**						
Infants 50 mg/m^2^	100	100	100	100	35.68	0
Infants 70 mg/m^2^	100	100	100	100	100	0
Children (age 2~11 years, 50 mg/m^2^)	100	100	100	100	43.75	0
Children (age 2~11 years, 70 mg/m^2^)	100	100	100	100	97.48	0.29
Adolescents (age 12~14 years, 50 mg/m^2^)	100	100	100	100	53.73	0.05
Adolescents (age 12~14 years, 70 mg/m^2^)	100	100	100	100	94.75	3.31
**Maintenance dose**						
Infants 40 mg/m^2^	100	100	100	100	9.99	0
Infants 50 mg/m^2^	100	100	100	100	65.21	0
Infants 60 mg/m^2^	100	100	100	100	97.03	0.01
Children 40 mg/m^2^	100	100	100	100	21.50	0
Children 50 mg/m^2^	100	100	100	100	62.57	0.05
Children 60 mg/m^2^	100	100	100	100	90.01	0.65
Adolescents 40 mg/m^2^	100	100	100	100	33.34	0.20
Adolescents 50 mg/m^2^	100	100	100	100	65.63	1.40
Adolescents 60 mg/m^2^	100	100	100	100	90.98	5.30

### Clinical Outcomes

In this study, 48 patients received caspofungin (loading dose of 70 mg/m^2^ followed by 50 mg/m^2^) as prophylaxis for fungal infections for a period of HSCT. The diagnosis of IFI mainly relied on a chest computed tomography (CT), clinical symptoms, blood culture, G test, and GM test. Patients were considered a probable IFI case with a chest CT scan positive for a halo or air crescent sign and large mass flocculent shadow. Routine blood culture, as long as the patients who receive bronchoscopy are all sent to receive bronchoalveolar lavage fluid culture meanwhile high throughput sequencing to find the pathogenic bacteria. In our study, most patients had a prophylaxis outcome (92%), only four patients with treatment failure had invasive pulmonary infection that diagnosed as IFI based on clinical symptoms combined with CT and bronchoalveolar lavage fluid (1,3-β-D-glucan) antigen test (G test) and Galactomannan test (GM test). The culture of bronchoalveolar lavage fluid was negative in three cases for consecutive two times and positive for *C*. *parapsilosis* in one case that was identified as *C. parapsilosis* infection (MIC = 0.5 mg/L). The median ± standard deviation of AUC_24_ in successful and failed cases group are 394.93 ± 91.14 mg·h/L and 352.08 ± 24.43 mg·h/L (t = 0.929, P = 0.358) at the loading dose, 300.37 ± 88.3 mg·h/L and 247.71 ± 33.67 mg·h/L (t = 1.175, P = 0.246) at the maintenance dose respectively. In this one failure case, the AUC_24_/MIC ratios at the loading dose and maintenance dose were 695.42 and 528.38, respectively. They were much lower than fungicidal target AUC_24_/MIC ratios ⩾ 1185 for *C. parapsilosis*. At the maintenance dose AUC_24_/MIC ratios were lower than the fungistatic target AUC_24_/MIC ratios ⩾ 559 for *C. parapsilosis*, which might lead to the failure of preventive treatment.

## Discussion

Caspofunign has been shown antifungal activity against *Candida* spp. and *Aspergillus* spp. *in vitro* and *in vivo*. The mechanism of action is noncompetitively inhibits β-(1,3)-D-glucan synthase enzyme that is necessary for the synthesis of the cell wall in many fungal species (Lewis, 2011). Antifungal prophylaxis is more important because of the high morbidity and mortality associated with IFIs, especially in immuno-compromised pediatric patients undergoing allo-HSCT (Morita et al., 2018). It was an effective strategy that patients who received allo-HSCT underwent primary antifungal prophylaxis with caspofungin (Mariotti et al., 2019).

A study indicated that body weight and total protein concentration were identified as covariates that affected Vc in critically ill patients on continuous venovenous hemodiafiltration (Aguilar G. et al., 2017; Perez-Pitarch et al., 2018). Others reported weight and disease state had significant effects on caspofungin PKs in pediatric patients (Li et al., 2011). Xin-Mei Yang studied the PK of caspofungin in children (2–12 years old). The median of CL and central volume distribution were 0.21 L/h/m^2^ and 2.23 L/m^2^, respectively. The final model was a two-compartment PK model and BSA had a significant correlation with caspofungin pharmacokinetics (Yang et al., 2019). We showed the quite similar results that CL was 0.19 L/h/m^2^ and V_d_ was 1.72 L/h/m^2^ and we tried to describe the pharmacokinetic process of caspofungin *in vivo* by using the one-or two compartment models. The OFV, Akaike's information criterion (AIC), and Bayesian information criterion (BIC) values of the one-compartment model are 603.1, 613.1, and 727.8, while two-compartment model are 593.6, 611.6, and 638.1. Compared with the two-compartment model, the OFV value is slightly lower, but the AIC and BIC values are roughly the same. In our studies, we obtained only two peak concentrations which made more individual variability in peripheral volume of distribution (v_2_) and inter-compartment clearance (Q), the distribution phase plasma concentration data collected are few, one-compartment PK model with first-order elimination best described the data. Among all covariates analyzed, from covariate screening procedure, BSA and AST can be indicated as significant optimal covariates for PK data modeling from the results of PopPK analysis and were included into the final model. Our analysis demonstrated that BSA had a significant effect on caspofungin clearance. The conclusion is in line with those reported by Zhao Wei et al. Therefore, the caspofungin dosage is administered on BSA adjusted (milligrams per square meter) would be rational for patients.

Caspofungin distributed well into tissues including the liver, lung, and spleen which exhibits antifungal activity in a concentration-dependent manner. Different studies have indicated that standard dosing regimens of caspofungin are appropriate in patients undergoing hemodiafiltration and continuous renal replacement therapy (Roger et al., 2017). Real-world studies or clinical trial on caspofungin population pharmacokinetics and the dosing regimen in pediatric population are limited. Nevertheless, undergoing allo-HSCT pediatric patients can result suboptimal outcomes owing to the special PK characteristics.

The manufacturer of caspofungin does not recommend dose adjustment in patients with mild hepatic dysfunction, whereas a lower maintenance dose of 35 mg daily after a loading dose of 70 mg is recommended in patients with moderate hepatic dysfunction (Song and Stevens, 2016). In this study, no significant difference could be identified in the caspofungin BSA-normalized CLs between mild hepatic dysfunction and normal hepatic dysfunction in pediatric patients. So dosage adjustment is not necessary in children with mild hepatic dysfunction. From the PopPK analysis and Monte-Carlo simulation test, we found that the existing standard doses of caspofungin (i.e. a loading dose of 70 mg/m^2^ followed by a maintenance dose of 50 mg/m^2^) is enough for fungal infection against with *C. albicans* and *C. glabrata* in all different age groups. For fungistatic target of *C. parapsilosis*: 70 mg/m^2^ at first dose, 60 mg/m^2^ of daily dose for pediatric patients likely had an optimal (PTA ⩾ 90%) success that predicted by the results of simulations. In addition, all of the regimens achieved low PTA against *C. parapsilosis* fungistatic target. For *C. albicans* with an MIC > 0.25 mg/L and *C. glabrata* with an MIC > 0.5 mg/L, recommended caspofungin dosage regimens cannot achieve PK/PD target. However, 70 mg/m^2^ maintenance dose followed by 70 mg/m^2^ loading dose led to sub-optimal therapeutic targets that for *C. parapsilosis* with MIC > 0.25 mg/L. Higher than the above doses can be required in allo-HSCT patients with infected *Candidiasis*.

Data from adult studies have shown that caspofungin has a well tolerated and exhibits antifungal activity in a dose-dependent manner (Cornely et al., 2011). Caspofungin can be used with the maximum dose for adults up to 200 mg/d from the previously published data (Maximova et al., 2017). Previously, in a murine model of systemic candidiasis, preclinical data from a dose-fractionation study of caspofungin indicated that AUC predicted efficacy (Shen et al., 2014). Caspofungin is an effective primary therapy for preventing IFI in the allo-HSCT recipients. In our research, most patients had a favorable outcome (92%), only four patients had invasive pulmonary infection that diagnosed as IFI. In summary, caspofungin exposure level *in vivo* was lower in treatment failed cases group but no significant difference in AUC was registered in the treatment success and failed subgroups. Caspofungin has an advantageous safety profile. In clinical trials, gastrointestinal side effects, elevations in alanine/aspartate aminotranferase levels representing are the most commonly adverse effects. Recent studies have demonstrated that when caspofungin co-administered with dexamethasone, efavirenz, carbamazepine, or phenytoin, the dosing should be increased. In the present study, 48 caspofungin recipients received standard-dose and we did not find drug-related adverse events. A larger sample size should be included in clinical further study to document clinical efficacy.

Our study had some limitations that need to be declared. We only focused on cases that children received allo-HSCT and the number of cases was limited, especially the number of cases with severe abnormal liver function was rare. The pharmacokinetic information of this group was less obtained. More studies are needed to identify the PK/PD targets in future.

## Conclusion

This paper presents the caspofungin PKs in pediatric patients aged 0.6–14 years with allo-HSCT and assessed the influence of demographic characteristics and clinical covariates on caspofungin disposition. BSA and AST were conformed to be determinants of caspofungin CL and V_d_ in our PopPK analysis. Dosage adjustment of caspofungin is not necessary in patients with mild liver dysfunction. From the analysis of the population pharmacokinetic and Monte-Carlo simulations, our results showed that 70 mg/m^2^ loading dose and 50 mg/m^2^ maintenance dose regimen can be recommended for a successful outcome against *C. albicans* and *C. glabrat*a with high probability, but focused on *C. parapsilosis*, conventional doses may not be sufficient to achieve optimal activity which provided theoretical basis approach for dosage individualization caspofungin use in pediatric patients.

## Data Availability Statement

All datasets generated for this study are included in the article/supplementary material.

## Ethics Statement

The studies involving human participants were reviewed and approved by the Ethics Committee of Wuhan Children’s Hospital (Wuhan Maternal and Child Healthcare Hospital), Tongji Medical Collage, Huazhong University of Science and Technology, Hubei, China. Written informed consent to participate in this study was provided by the participants' legal guardian/next of kin.

## Author Contributions

C-HN and YW designed and supervised the clinical trial. HX, L-LG, Y-MN, L-PX, and L-PY carried out the clinical trial. S-LW conducted the population pharmacokinetic analysis. C-HN and YW wrote the manuscript. L-PY and C-HN edited the manuscript.

## Funding

This work was financially supported by the Youth Program of National Natural Science Foundation of China (Grant Agreement Number: 81600123).

## Conflict of Interest

The authors declare that the research was conducted in the absence of any commercial or financial relationships that could be construed as a potential conflict of interest.
